# The Assessment of Undergraduate Medical Students’ Satisfaction Levels With the Objective Structured Clinical Examination

**DOI:** 10.5812/ircmj.13088

**Published:** 2014-08-05

**Authors:** Ahmad Khosravi Khorashad, Somayyeh Salari, Humain Baharvahdat, Sepideh Hejazi, Shiva M Lari, Maasoomeh Salari, Maryam Mazloomi, Shahrzad M Lari

**Affiliations:** 1 Department of Internal Medicine, Mashhad University of Medical Sciences, Mashhad, IR Iran; 2 Department of Neurosurgery, Mashhad University of Medical Sciences, Mashhad, IR Iran; 3 Health Care Administration, California State University of Northridge, Northridge, USA; 4 COPD Research Center, School of Medicine, Mashhad University of Medical Sciences, Mashhad, IR Iran

**Keywords:** Undergraduate, Medical Students, Internal Medicine, Personal Satisfaction

## Abstract

**Background:**

The objective structured clinical examination (OSCE) has been introduced as an efficient method for the assessment of medical students.

**Objectives:**

The aim of the present study was to determine the satisfaction level of undergraduate medical students of internal medicine department with the OSCE.

**Materials and Methods:**

This was a descriptive cross-sectional study, performed on all available undergraduate students at the end of their internal medicine training period in Mashhad University of Medical Sciences. The students responded to 15 multiple-choice questions with confirmed validity and reliability.

**Results:**

The majority of the students (94.5%) had a positive attitude toward the OSCE and mentioned that the OSCE format was a more appropriate type of exam than other methods of testing; however, 79.1% thought that the OSCE format was stressful. In addition, the participants’ sex had no effect on their level of satisfaction with the examination. Likewise, there was no significant correlation between their level of satisfaction and their age, marital status, or lack of previous experience with this type of exam.

**Conclusions:**

If the exam standards are met and a uniform dispersion of the scientific content is maintained, the OSCE method of assessment can be recommended as an efficient and applicable method for assessing medical students.

## 1. Background

The evaluation of the medical students’ ability can appropriately reflect the efficacy of the educational curriculum before graduation and lead to a better educational process (1, 2). There are different methods for the evaluation of practical skills acquisition, one of which is Objective Structured Clinical Examination (OSCE).

OSCE was introduced in 1975 by Harden for evaluating the medical students’ clinical competency (2, 3). OSCE has become an excellent tool for evaluating several aspects of students’ clinical skills (4) and its validity and reliability in the fields of medicine have been established in many studies (5-8). OSCE is a clinical demonstration that improves the students’ performance and strengthens their professional role (9). The OSCE has been proven to be a technically feasible and authentic evaluation method that yields valuable information for decisions regarding student performance, faculty teaching, and curriculum planning (10).

On this exam, wide ranges of technical and basic skills are evaluated through a simulated environment rather than the real patient, with the same test subject and questions for all students. Typically, the students rotate through the five- to 20-minute standardized stations during the OSCE for performing the requested skills (9-11).

As a modern type of examination, it has important advantages over oral ones since it is a performance-based assessment exam (9). According to previous studies, this exam lacks the deficiencies seen in unstructured oral exams such as unfairness and low validity and reliability and can test for a wide range of skills.

Therefore, given the global success of the OSCE, measures should be taken to improve the quality of the exam to a satisfactory level (4). One of the main OSCE-accompanying problems is increased students' anxiety. Faryabi et al. showed that the increased student stress in association with the OSCE format could influence their objection to the continuation of this type of an assessment, with only 34.8% considering the OSCE an educationally-advantageous exam (12).

## 2. Objectives

This study aimed to determine the level of satisfaction of the undergraduate medical students of internal medicine at Ghaem Hospital, Mashhad, Iran, in order to detect such problems and contribute to the improvement of OSCE.

## 3. Materials and Methods

### 3.1. Participants

This cross-sectional study was conducted from February 2012 to February 2013 by the Internal Medicine Educational Group of Ghaem Hospital, Mashhad University of Medical Sciences, Mashhad, Iran, in order to assess the level of satisfaction of the undergraduate (at the fourth year of study) medical students in department of internal medicine. The period of clerkship was three months. All undergraduate medical students in the final stages of their internal medicine clerkship were enrolled in this study. The students were excluded if they did not tend to participate in this study. All participants gave their written informed consent for participating in this study. The Ethics Committee of Internal Medicine Department approved the study protocol.

### 3.2. The Exam

The students' performance was evaluated using an OSCE format consisting of 13 stations covering all areas of internal medicine contents and through interviews with simulated patient (SP), clinical examination, practical skills, communication with patients, and education of patients. A time limit of five minutes was set for each station, at the end of which students had to leave the given station. The total examination time was 80 minutes.

### 3.3. Evaluation of Satisfaction Level

At the end of the examination, a questionnaire concerning the satisfaction level of students from OSCE was handed out to the students. The questionnaire was previously designed by Movaffaghi et al. for the evaluation of the residents of medicine and was confirmed in terms of content validity (by expert judgment) and had a reliability of 88% using the Cronbach's alpha coefficient of internal consistency (13). The questionnaire was designed as closed-response questions and consisted of 15 questions. The four possible choices were completely disagree (25 scores), disagree (50 scores), agree (75 scores), and completely agree (100 scores); in the data analysis, the former two choices were considered as negative and the latter two as positive opinions.

### 3.4. Data Analysis

After data collection, coding and analysis were done using the SPSS version 11.5 (SPSS Inc, Chicago, IL, USA). Descriptive statistics were used to summarize the demographic characteristics of the items. The data of ordinal variables were presented as median, mode, and range. Spearman correlation coefficients were calculated. A P values < 0.05 was considered as statistically significant.

## 4. Results

A total of 76 undergraduate medical students were enrolled in this study. Four questionnaires were deleted because of incomplete responses. Finally, 72 questionnaires were analyzed. The male to female ratio was 1:2 (24 males and 48 females). Approximately one-third of them (36%) were married. Overall, 63% had no previous experience with an OSCE format exam. The mode, median, and range of scores of each item in questionnaire are shown in Table 1. The majority of the students (94.5%) had a positive attitude toward OSCE and mentioned that this format is a more appropriate type of exam than other methods of testing. More than two-thirds of the students (72.2%) believed that the OSCE was comprehensive and covered all the undergraduate studentship educational curricula. Most of the participants (91.7%) responded positively to the question of whether the OSCE can assess practical skills that cannot be tested through written exams.

**Table 1.** The Descriptive Statics of Evaluated Factors About OSCE ^a^

Factors Related to OSCE	Median	Mode	Range
**Appropriateness **	75	75	25-100
**Assessment of Clinical Skills**	75	75	25-100
**Comprehensiveness**	75	75	50-100
**Assessment of Practical Skills**	75	75	25-100
**Response to Scientific Requirements**	75	75	0-100
**Matching of Stations’ Content With the Scientific Level of Students**	75	75	25-100
**Appropriate Simulation of Clinical Environment**	75	75	0-100
**Proper Guideline**	75	75	0-100
**Sufficient Time**	75	75	0-100
**Appropriate Number of Stations**	75	75	25-100
**Cooperation of Simulated Patient **	75	75	0-100
**Appropriate Supervision Of Stations**	75	75	25-100
**Appropriate Information About Exam**	75	75	0-100
**Stressfulness **	75	75	25-100
**Improvement of Students’ Practical Skills**	75	100	25-100

^a^ Abbreviation: OSCE**,** objective structured clinical examination.

Totally, 75% of the students advocated that the OSCE tested areas of knowledge, one’s perspective, and practical skills. Furthermore, most participants felt that the exam had met the scientific requirements (73.6%), the questions matched their level of understanding (83.4%), the stations were appropriate simulations of the clinical environment (76.4%), the examination time was sufficient (75%), the stations were appropriately designed (91.6%), simulated patients had good cooperation (72.2%), stations were well supervised (94.5%), and they were well informed about the exam (88.9%). In fact, less than 50% of participants responded negatively to the questions.

**Figure 1.**
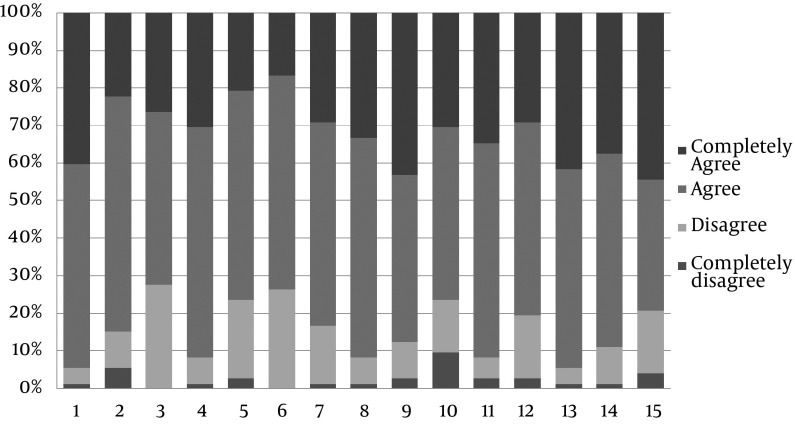
The Frequency of Students’ Response to Each Item of Questionnaire

However, 79.1% thought that the OSCE format was stressful. Overall, 87.5% of the students considered the OSCE as an appropriate method for the improvement of their practical knowledge. In Figure 1, the frequency of students’ response to each item of questionnaire is illustrated.

In the present study, the participants’ sex had no effect on their level of satisfaction with the examination. Likewise, there was no significant correlation between their level of satisfaction and their age, marital status, or lack of previous experience with this type of an exam (r = 0.2, P = 0.7; r = 0.3, P = 0.1; and r = 0.2, P = 0.8, respectively).

## 5. Discussion

Given the efficiency of the OSCE in the clinical assessment of medical students, the present study was designed to assess the level of satisfaction with this type of exam amongst the undergraduate medical students of internal medicine. The most frequent positive response belonged to the question of whether the OSCE was a more appropriate method of assessment than other exam formats, while the students were least satisfied with the simulated patients’ cooperation.

In this study, 94.5% of the participants perceived that the OSCE was the more appropriate type of assessment. This finding was in agreement with other studies that showed the OSCE method of assessment is preferred to the more conventional formats and is fair in comparison to the multiple-choice questions and oral exams (1, 14, 15). Almost 92% of the participants believed that the OSCE could assess practical skills, which could not be tested via written exams. This finding was in agreement with that of Bolahari et al. who considered the OSCE as an efficient way of assessing practical skills and qualifications (16).

Totally, 75% of the undergraduate students were convinced that the OSCE had covered the main areas of education, i.e. knowledge, perspective, and practical skills. This was similar to the findings based on the hypothesis of an association between knowledge and clinical skills (17); however, it was in contrary to the conclusion made by Bolahari et al. (16) stating that in comparison with other exams such as those with multiple-choice questions, the OSCE was not an appropriate way of evaluating one’s level of knowledge.

One of the main problems with the OSCE is the stressful environment of the exam. Allen et al. showed that although the medical students had high levels of confidence in their abilities during the requested skills through the OSCE, they were nervous (18). In addition, Pierre et al. showed that students believed that OSCE was more stressful than other exams (1). According to different studies, there are various reasons for such stress including the lack of familiarity with the examination, the presence of supervisors, and nonstandard questions. In our study, approximately 80% of undergraduate students felt that this type of an exam had induced stress, a finding that was supported by previous studies (12, 19, 20). However, Jalili et al. (21) showed that 63.3% of medical students of Kerman University of Medical Sciences had rejected the notion that the OSCE induced any extra stress; the same was reported by Hoseini et al. (22).

Some limitations need to be acknowledged and addressed regarding the present study. Firstly, it was a cross-sectional study in a clinical ward; similar studies on the OSCE in other clinical wards may assist in the final comparisons and conclusions. Secondly, considering the standards of the OSCE, reevaluation with an increased number of stations is recommended. Thirdly, due to small sample size, we could not generalize our findings to target population. Similar studies with larger sample for more expandable results are recommended.

We studied the satisfaction levels of undergraduate students of internal medicine with the OSCE. In the present study, the majority of the students believed that the OSCE was superior to other assessment formats. Finally, correct training and the better selection of patient actors can help to strengthen the examination.
